# Raising awareness of carrier testing for hereditary haemoglobinopathies in high-risk ethnic groups in the Netherlands: a pilot study among the general public and primary care providers

**DOI:** 10.1186/1471-2458-9-338

**Published:** 2009-09-15

**Authors:** Stephanie S Weinreich, Elly SM de Lange-de Klerk, Frank Rijmen, Martina C Cornel, Marja de Kinderen, Anne Marie C Plass

**Affiliations:** 1 VU University Medical Center, Department of Clinical Genetics, Community Genetics, Amsterdam, the Netherlands; 2 EMGO Institute for Health and Care Research, VU University Medical Center, Amsterdam, the Netherlands; 3 VU University Medical Center, Department of Clinical Epidemiology and Biostatistics, Amsterdam, the Netherlands; 4 Foundation Erfocentrum, Woerden, the Netherlands

## Abstract

**Background:**

In the Netherlands no formal recommendations exist concerning preconceptional or antenatal testing for carriership of hereditary haemoglobinopathies. Those at highest risk may be unaware of the possibility of carrier screening. While universal newborn screening has recently been introduced, neither preconceptional nor antenatal carrier testing is routinely offered by health care services to the general public. A municipal health service and a foundation for public information on medical genetics undertook a pilot project with the aim of increasing knowledge and encouraging informed choice. Two groups were targeted: members of the public from ethnic groups at increased risk, and primary health care providers. This study examines the effectiveness of culturally specific 'infotainment' to inform high-risk ethnic groups about their increased risk for haemoglobinopathies. In addition, the study explores attitudes and intentions of primary care providers towards haemoglobinopathy carrier testing of their patients from high-risk ethnic groups.

**Methods:**

Informational sessions tailored to the public or professionals were organised in Amsterdam, and evaluated for their effect. Psychological parameters were measured using structured questionnaires based on the Theory of Planned Behaviour.

**Results:**

The pre-test/post-test questionnaire showed that members of the public gained understanding of inheritance and carriership of haemoglobinopathies from the "infotainment" session (p < 0.01). Perceived behavioural control, i.e. the feeling that they could actually get tested if they wanted to, increased in the targeted age group of 18-45 years (N = 41; p < 0.05). 191 surveys were collected from general practitioners or midwives. Their attitude towards the education programme for high-risk ethnic groups was positive, yet they did not show strong intention to effectuate carrier testing of their patients on the basis of ethnicity. The main factor which explained their (lack of) intention was social norm, i.e. their perception of negative peer opinion (41% variance explained). The majority of primary health care providers felt that policy change was unnecessary.

**Conclusion:**

The "infotainment" programme may have a positive effect on people from high-risk groups, but informed general practitioners and midwives were reluctant to facilitate their patients' getting tested. Additional initiatives are needed to motivate primary care providers to facilitate haemoglobinopathy carrier testing for their patients from high-risk backgrounds.

## Background

It has been estimated that there are currently some 140,000 carriers of hereditary haemoglobinopathies (i.e. sickle cell anaemia and thalassaemia) living in the Netherlands. A large proportion, about 100,000 (70%), are immigrants or second generation descendants of immigrants from areas with high carriership frequencies [1,2]. Carriers are usually unaffected themselves. The birth prevalence of hereditary haemoglobinopathies in the Netherlands has been estimated at 60 per year, with about 80% cases of sickle cell disease and 20% of β-thalassaemia [3]. These figures were confirmed by preliminary results in 2007, the year in which universal newborn screening for sickle cell disease was introduced [4].

The World Health Organisation (WHO) has recommended developing services which integrate treatment, carrier detection, and genetic counselling for sickle cell disease [5,6]. For some time now there has been discussion in the Netherlands about whether carriership testing for e.g. haemoglobinopathies should be introduced, and if so, how [7-10]. Strategies could be universal or selective, and could be aimed at preconceptional testing of couples [11], antenatal testing of pregnant women (followed by partner screening and prenatal diagnosis of the child) [12], and/or neonatal screening. However, at the time of writing, no formal recommendations from Dutch public health authorities have been made for preconceptional or antenatal testing of asymptomatic persons [13,14], nor is there any systematic provision of information for the general public. In 2005 the Health Council of the Netherlands recommended expanding the newborn screening programme to include sickle cell disease [15]. This is because early diagnosis and treatment has health benefits for affected children. Besides identifying patients, newborn screening also results in unsought identification of haemoglobinopathy carriers. Although parents may opt out of receiving this information, those who do learn that their child is a carrier have to cope with the genetic implications for themselves, the child, and other family members. It is likely that requests for 'cascade' carrier testing will ensue. In addition, increased awareness in society at large may lead to more frequent requests for preconceptional carrier testing. Primary care providers, in addition to paediatric and clinical genetic specialists, will be called upon to meet these needs.

In countries where a minority of the population has a recognised elevated risk for a genetic disease, an offer of carrier screening on the basis of ethnicity or ancestry can be a sensitive issue. Nevertheless, the American College of Obstetricians and Gynecologists recommends that carrier screening be offered to individuals of ancestry with higher risk [16]. In England, the NHS Sickle Cell and Thalassaemia Screening Programme includes antenatal screening to facilitate informed reproductive choices to women and couples identified as having a higher genetic risk for hereditary haemoglobinopathies [17]. Interestingly, a plea has been made for a more active role for primary care givers, because this would lead to more people being identified in time, enabling them to make a truly informed choice [5].

Carrier testing has potential harms of psychological burden, stigmatisation and discrimination. However, it confers benefits on couples at risk by giving them reproductive choices. In addition, carrier testing can help minimise delay in diagnosis and treatment of anaemic children. A more active policy for carrier testing could thus lead to health benefits, although this would add to the workload of certain healthcare professionals. In the Netherlands, each community-dwelling person is registered with a general practitioner (GP), and patients have to consult their GP before entering the rest of the medical system. Carriership of haemoglobinopathies can often be ascertained by primary care providers (i.e. GPs or midwives) on the basis of simple haematological and biochemical testing. Additionally, centres for clinical genetics and specialised laboratories are needed for prenatal diagnosis, for carriership testing for α-thalassaemia and for testing of complex cases, partners of carriers and high-risk couples [2].

At present, many first- and second-generation immigrants in the Netherlands with a high risk for hereditary haemoglobinopathies are probably unaware of the possibility for carrier testing [18]. While anaemia and a family history of haemoglobinopathy are recognised indications for diagnostic haemoglobinopathy testing of patients from high-risk ethnic groups for primary care providers [19], no guidelines are available on testing non-symptomatic patients solely on the basis of ethnicity. Before measures can be proposed to increase support for carrier testing, it may be helpful to explore the current knowledge, attitudes and practice among primary care providers as well as the population with an increased risk.

Amsterdam was chosen as the area for this pilot project, since at least 25% of the current inhabitants were born- or have ancestors who were born- in areas with relatively high frequencies of carriership for hereditary haemoglobinopathies, i.e. Surinam, the Antilles, Turkey, Morocco, and western Africa [20]. The first major aim of the study was to measure the effect of a culturally specific 'infotainment' session about hereditary haemoglobinopathies on knowledge, attitudes and intentions to get tested among members of the public from high-risk ethnic groups, especially persons of reproductive age (18-45). The choice of 'infotainment' with culturally specific elements was based on the findings of an earlier project in the Netherlands concerning informing people of Turkish and Moroccan background about hereditary and congenital disorders (VSOP, the Dutch Genetic Alliance, 2003). Moreover, the Amsterdam Municipal Health Service had experience with culturally specific health education projects [21,22]. The other main focus of the study concerned primary care providers, i.e. general practitioners (GPs) and midwives. The first aim was to measure their attitudes to current initiatives towards informing the population about increased risk. The second aim was to measure primary care providers' willingness to effectuate carrier testing for their patients on the basis of ethnicity. Finally, the effect of an educational session for primary care providers was explored. The analyses were based on the Theory of Planned Behaviour [23], a well-known psychometric framework for studying determinants of health-related behaviour.

## Methods

### Participants and procedure

#### A. High-risk groups

Two districts of Amsterdam with a high percentage of immigrant residents were chosen as target areas. Members of the public from high-risk ethnic groups were recruited through a city-wide public relations campaign carried out by the company MCA Communicatie BV, Utrecht and Erfocentrum. Various media announced that bilingual meetings would be held. The sessions, organised in 2005 by the municipal health service (GGD Amsterdam) and Erfocentrum, included a play about sickle cell disease and thalassaemia, a panel discussion, various performances, and food. Verbal translation was provided or occurred informally. These sessions were held in the target neighbourhoods described above. In total, five bilingual sessions were held in Dutch plus a foreign language, i.e. one each in Papiamentu, Sranan Tongo, English, and Turkish, and two in Moroccan Arabic (for men and women, separately). Participants were asked to fill in questionnaires before and after the session. An impression of the play is available on the internet [24].

#### B. Primary care providers

All 410 general practitioners and all 24 midwifery group practices in Amsterdam were sent a questionnaire by post, including an announcement for an accredited educational session. A reminder was sent 3 weeks later. Two educational sessions were later held during the time frame of this study (2005-2006).

### Measures: Structured questionnaire

Structured questionnaires based on the Theory of Planned Behaviour [23] were used for both the high-risk group and the primary care providers.

#### A. High-risk groups

The questionnaire for the high-risk group was in Dutch. Pre- and post-test questionnaires were linked by a code and were identical, except that demographic questions were only asked once. Briefly, the questionnaire was intended to measure knowledge, attitude, social norm, perceived behavioural control, and intention to undergo screening. Ordinal items were measured on a 5-point scale. Details of the questionnaire are given in Additional File 1.

#### B. Primary care providers

In the postal questionnaire sent to them, primary care providers could indicate whether they wished to attend an educational session on a specific date. In the questionnaires used after the educational sessions, this question was replaced by 'Is this the first time you have completed this survey?'. These questionnaires were not paired.

The questionnaire for health care providers measured attitude, social norm, perceived behavioural control, intention to effectuate screening, current behaviour and opinion about policy. Ordinal items were measured on a 7-point scale. Additional File 2 provides details of this questionnaire.

### Analysis

All questionnaire data were analysed in SPSS 12. For each determinant which was measured by multiple questions, internal consistency was determined by calculating Cronbach's alpha, including the scale if any item were deleted. Exploratory factor analysis was done for determinants with three or more questions by unweighted least squares and oblimin rotation. Two-tailed values for Pearson correlation are reported.

Ordinal and continuous data obtained from the general public are reported as means. The effect of educational sessions for the general public was determined by paired T-tests. For primary care providers, where questionnaires were not paired, medians are reported because most of the data were not normally distributed. Differences between groups were assessed with the Mann-Whitney U test for unpaired ordinal variables, by the Wilcoxon signed ranks test for paired ordinal variables, and by the chi-squared test for dichotomous variables. Hierarchical linear regression was applied stepwise in order to analyse data obtained according to the Theory of Planned Behaviour. Effect modification was tested by adding interaction terms in the second block. In all cases, two-tailed p-values below 0.05 are reported as statistically significant.

### Informed consent

According to Dutch law this type of questionnaire research does not require approval from an Ethical Review Board.

## Results

### High-risk ethnic groups

#### Participants

80 attendees returned at least one questionnaire. Ethnicity was distributed as follows: 28% Surinamese, 24% Antillean, 16% Moroccan, 9% Turkish, 5% Dutch, 4% African, and 15% missing. Because the individual ethnic groups were represented by small numbers, most of the further analyses are not broken down by ethnicity. Details of meeting attendance and ethnicity of responders are shown in Table 1. In general the background of the attendees corresponded with the targeted group.

**Table 1:** Attendance and questionnaire response at public educational sessions

	**Language at session (besides Dutch)**					
	
	**Papiamentu**	**Sranan** **Tongo**	**English**	**Turkish**	**Moroccan** **Arabic**	**Total**
No. of attendees	23	125	30	40	56	**274**

Self-reported ethnicity of respondents*						
*Antillian*	19					19
*Surinamese*		21		1		22
*African*			3			3
*Turkish*			1	6		7
Moroccan**			1	2	10	13
*Dutch*		1		1	2	4
*unknown*	1	3	2	5	1	12

No. of questionnaires completed*	20	25	7	15	13	**80**

*At least one questionnaire filled in i.e. before and/or after.

**One person born in Algeria with both parents born in Morocco was classified under Moroccan ethnicity.

The mean age of participants was 42 years (range 18-77 years; 9 missing). Forty-one of the participants (58%) were in the targeted age group of 18 through 45 years, while 30 (42%) were older (9 missing). Gender distribution was 57 (79%) female, 15 (21%) male (8 missing). Forty-six of the participants (66%) had children, 24 (34%) did not (10 missing). The median number of children was 2 (range 0-8). Two (3%) participants or their partners were pregnant. There were no significant differences in gender or number of children among responders from the various ethnic groups. Age of respondents was almost significantly different among the groups (F(5,61) = 2.32; p = 0.05). The highest completed level of education was distributed as follows: 32% low, 42% intermediate and 26% high (11 missing). Due to the limited number of subjects in this study, the analyses do not include stratification or correction for demographic variables.

#### Effect of infotainment session on knowledge and psychological parameters

Prior to the educational session, about a quarter of respondents had never heard of the diseases in question, more than a third had never heard about carriership, and more than half did not know about carriership testing.

Understanding inheritance and carriership of haemoglobinopathies was tested in paired questionnaires before and after the session, with a maximum sum score of 3. The target group (N = 31) increased in mean score from 1.35 before to 1.55 after (NS), while the whole group (N = 59) increased from 1.15 to 1.46, mean increase 0.31, 95% CI = 0.09-0.52, p < 0.01.

Attitude towards information about carriership testing showed good consistency between the eight items (Cronbach's alpha 0.8). Two factors were extracted. Both the target group (N = 31) and the whole group (N = 57) had very high mean attitudes towards information about testing, respectively 4.4 and 4.5. After the educational session, there was no significant change in either group. Attitudes to participation in carrier testing showed good consistency between the eight items (Cronbach's alpha 0.8). Both the target group (N = 31) and the whole group (N = 58) had high mean attitudes towards participation in testing, respectively 4.3 and 4.5. There was no significant change in either group after the educational session.

The social norm scale showed high consistency (Cronbach's alpha 0.8). Mean scores were close to neutral, at 3.4 in both the target group (N = 31) and the whole group (N = 55). There was no significant change in either group after the educational session.

The scale for perceived behavioural control (pbc) showed only fair consistency (Cronbach's alpha 0.5), but this is in agreement with what is reported elsewhere [16]. Consistency was not substantially improved by removing questions. A single factor was extracted. The target group (N = 31) and the whole group (N = 56) had fairly positive mean scores for pbc (3.8). After the educational session, the target group (N = 31) had a significantly higher mean score for pbc, namely 4.0, mean increase 0.28, 95% CI 0.01-0.55, p < 0.05. The group as a whole also scored higher after the educational session, but this did not reach statistical significance.

The two questions relating to intention had poor consistency, and were treated separately. Intention to get tested scored high with a mean of 4.6 in the target group (4.3 in the whole group). Intention to prevent the birth of an affected child had a rather high mean score of 4.3 in the target group (4.2 in the whole group). Neither parameter changed significantly after the educational session.

On average, members of the target group (N = 31) felt fairly neutral about their personal risk of being a carrier with a score of 3.2, similar to the whole group (N = 56) at 3.0. There was no significant change in either group after the educational session. With regard to the effect of carriership on the decision to have children, the target group (N = 31) scored close to neutral at 2.7 (total group 3.0, N = 56). There were no significant changes after the educational session.

Three questions were posed regarding the burden of being a carrier. Cronbach's alpha was 0.7 showing fair consistency. Deleting the item about feeling less healthy increased Cronbach's alpha to 0.8. The remaining items, i.e. being treated differently or being discriminated, relate most closely to stigmatisation. Thus the mean score of these two items was further considered as a scale for stigmatisation. The target group (N= 31) scored 2.0, thus rather disagreeing that there would be stigmatisation (whole group N = 57, mean 2.2). With respect to feeling less healthy if a carrier, the target group (N = 31) was close to neutral (mean 2.7; total group N= 57, mean 3.0). There were no significant changes after the educational session either on the stigmatisation scale or on feeling less healthy.

The scale for equal access to care showed fair consistency (Cronbach's alpha 0.7). The target group (N= 31) as well as the whole group (= 56) were very positive about equal access to care (mean 4.5 for both). There was no significant change after the educational session.

### Primary care providers

#### Participants

**Figure 1:**
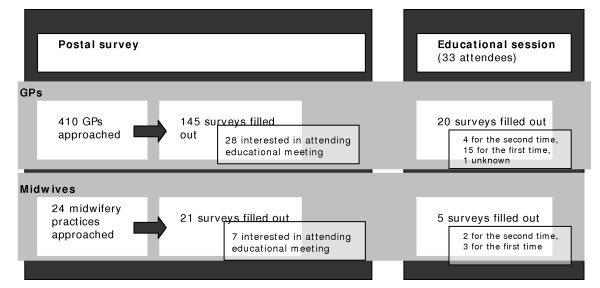
**Flow diagram for survey of primary care providers**.

Primary care providers were surveyed via a postal questionnaire; later, participants in an educational meeting were also surveyed. There was some overlap between the two groups, but pairing of questionnaires was not possible. Persons who possibly responded twice were excluded from the analysis of attendees, except when the attendees were compared as a group to those who had not (yet) attended a session. 160 and 24 definitely unique questionnaires were filled in by GPs and midwives, respectively (Figure 1).

#### Psychological parameters and current practice

Attitude was measured by six questions, which showed good consistency (Cronbach's alpha 0.8). Two factors were extracted. The psychological parameters and current practice are reported in Table 2. Note the 7-point scales, in contrast to the 5-point scales used in the questionnaire for high-risk groups. Both professional groups had fairly positive scores on attitude towards the current initiatives relating to carrier testing. They felt neutral about social norm, i.e. what they imagine their colleagues' opinion to be. Primary care providers perceived fair to good behavioural control of their ability to effectuate carrier testing for their patients. The intention to effectuate carrier testing solely on the basis of ethnicity scored as neutral or slightly more certain than neutral.

**Table 2:** Comparison of parameters by profession (medians)

		**GPs**^**1**^	**midwives**^**2**^
Psychological parameters	attitude	5.0	5.7
	
	social norm	4.0	4.0
	
	perceived behavioural control	5.0	6.0
	
	intention	4.0	4.5

Current testing for HbP carriership	based on ethnicity	4.0	5.0
	
	based on ethnicity + anaemia	5.0	6.0*

^1^N = 156 with no more than 8 missing per variable.

^2 ^N = 24 with no more than 3 missing per variable.

*Mann-Whitney U test p < 0.001

Current facilitation of patients requesting carrier testing solely on the basis of ethnicity was reported as neutral or slightly more often than neutral, by GPs and midwives respectively. Regarding referral of anaemic patients for carrier testing, solely on the basis of ethnicity, GPs reported that they do so rather often while midwives reported doing so very often. In this case the difference between the two professional groups was statistically significant (median GPs 5.0, midwives 6.0, Mann-Whitney U test p < 0.001). On most of the other items the midwives also scored more positively than GPs, though this did not reach statistical significance. Table 2 gives an indication that all primary care providers are more prone to effectuating carrier testing for their anaemic patients for haemoglobinopathy carrier testing, than they are to refer patients who request this carrier testing solely on the basis of their ethnic background. When all primary care providers were pooled, willingness to refer anaemic patients was significantly higher than willingness to refer non-symptomatic patients for carrier screening (median 5.0 for anaemic patients vs. 4.0 for the general case, Wilcoxon signed ranks p < 0.001).

#### Ethnic make-up of practices

Primary care providers were asked to estimate what percentage of their patients are from ethnic minorities. There was a wide distribution of this proportion (mean 38.7%, SD 26.4%). The percentage of ethnic minority patients in a practice did not significantly correlate with the primary care providers' attitude, social norm, perceived behavioural control, intention, or current practice in referring anaemic patients. Interestingly, there was a weak but significant positive correlation between the percentage of patients from ethnic minorities in a practice, and how prone the primary care providers are to refer patients who request carrier testing on the basis of ethnicity (r = 0.20, p < 0.05).

#### Need for policy change

Of all primary care providers, 60% did not think that policy change was needed for advising patients to get carrier testing on the basis of ethnicity (N = 166).

#### Explaining intention

Pearson correlation was calculated between the determinants of the theory of planned behaviour (Table 3). Perceived behavioural control was weakly correlated with intention, while strong correlations were found between attitude and intention, social norm and perceived behavioural control, and social norm and intention. Hierarchical linear regression was performed stepwise with attitude, social norm and perceived behavioural control in the first step. Social norm was found to be a very strong predictor of intention, explaining 41% of the variance (multivariate B = 0.70, p < 0.001) while attitude increased the explained variance by another 7% (B = 0.41, p < 0.001). There was no evidence for interaction between social norm and attitude.

**Table 3:** Correlation between determinants from the theory of planned behaviour in primary health care providers

	**attitude**	**social** **norm**	**pbc**	**intention**
attitude		0.15	0.10	0.35**
social norm			0.22**	0.65**
pbc intention				0.18*

pbc = perceived behavioural control

* = significant at 0.05 level

** = significant at 0.01 level

#### Educational session

The educational session was considered in two ways. First, psychological parameters and current referral practice were compared between primary care providers who were interested in attending the educational session and those who did not express this interest. Only one statistically significant difference was found: primary care providers who were interested in attending the educational session were more prone to do carrier testing on the basis of ethnicity than those who were not interested in attending (median 5.0 vs. 4.0, Mann-Whitney U test p < 0.05). The effect of the educational session could not be measured directly because paired questionnaires could not be identified. However, on a group level a comparison was made between primary care providers who filled in postal questionnaires and primary care providers who filled in the questionnaire after the educational session. There were no significant differences between the groups with respect to psychological parameters or current referral practice (Mann-Whitney U).

## Discussion

In this study, about one quarter of the respondents from high-risk backgrounds had never heard of hereditary haemoglobinopathy (i.e. sickle cell anaemia and thalassaemia). This figure is probably an overestimate of familiarity with the subject in the general population. In an unpublished, face-to-face survey held in 2005, 60% or more of members of several ethnic minorities had never heard of hereditary haemoglobinopathies [unpublished report, MCA Communicatie BV. Bekendheid erfelijke bloedarmoede onder Turken, Marokkanen, Surinamers, Antillianen en Nederlanders [Familiarity with hereditary haemoglobinopathy among Turkish, Moroccan, Surinamese, Antillian and Dutch people] 2005]. Through the infotainment session, understanding of haemoglobinopathies increased. The target group, consisting of individuals in the reproductive age-group, showed a significant increase in perceived behavioural control. This is encouraging because this is the group which might be faced with reproductive choices, should they get screened. Increased perception of control has been described before as a goal, and indeed a result of individual genetic counselling [25]. It is also encouraging that the infotainment session did not lead to an increase in risk perception or more negative feelings about the burden of carriership. Moreover, the possibility of stigmatisation was no issue to the participants; they were strongly in favor of equal access to carrier testing and information about carrier testing. These positive results are in accordance with a recent study which showed that an offer of preconceptional, ancestry-based carrier screening for cystic fibrosis and/or hereditary haemoglobinopathies by GPs was evaluated positively by members of the public in Amsterdam [26].

Primary care providers clearly approved of patient-education programmes informing the Dutch migrant population about their increased risk and carrier screening for haemoglobinopathies, but showed reserve about effectuating haemoglobinopathy carrier testing solely on the basis of ethnicity. This contrasts with the fact that they do take ethnicity into account, when diagnosing anaemic patients. The same attitudes and practices were found among primary care providers who attended an educational session on this subject. Although the prospect of attending an educational session appealed most to primary care providers who were already convinced that carrier testing on the basis of ethnicity is a good idea, no evidence was found that primary care providers who actually did attend scored higher on any of the measured parameters. However, a paired pre-test/post-test design, stratified for profession, would be more informative for measuring the effect of the educational session. The trend that midwives scored more positively than GPs in all measured parameters should be investigated further. Intention to refer patients for carrier testing solely on the basis of ethnicity was mainly predicted by the perception of colleagues' opinion (social norm). However, the majority of health care providers did not feel that policy change was needed with respect to their own effectuation of carrier screening. This discrepancy echoes the recent analysis by Achterbergh et al., who argued that effective implementation of preconceptional screening for hereditary haemoglobinopathies in the Netherlands will require changes among various stakeholders, at several levels of society [13]. The theory of planned behaviour explained 49% of variance in intention, which is at the high end of the range of what can be achieved by this method [27].

This study has some limitations. Attendees were self-selected and may not be representative of the population of interest. Positive selection bias of the respondents is very plausible; people who took the trouble to attend an infotainment session probably considered treatment and/or prevention of disease to be both worthwhile and desirable. On the other hand, the participants turned out not to be familiar with the subject at the start of the session. The small number of completed questionnaires at the Turkish meeting was partly due to unclear instructions given by the organisers. In general, participants may have failed to fill in the questionnaire because of insufficient grasp of written Dutch. This might limit the ascertainment of attitudes of recent immigrants or persons with a low educational level. Future research should compare the effectivity of infotainment to other types of communication, in helping people to make an informed choice.

The survey of primary care providers had better external validity. The response rate to the first survey was very high for midwives' practices, namely 21 out of 24. However, only 145 out of 410 GPs responded. It is striking that in another recent study in Amsterdam on haemoglobinopathy carrier screening, only 20% of the GPs approached agreed to participate, even though they all had their practices in districts with a high percentage of non-Western immigrants [11]. However, in the absence of comparative information about responders and non-responders in our study, selection bias cannot be excluded. Finally, some GPs commented on the use of the word "referral" in the questionnaire. Since they usually diagnose haemoglobinopathy on the basis of lab tests, which they order themselves, without sending the patient to another medical specialist, they found the word "referral" inappropriate or confusing.

## Conclusion

The patient-education infotainment session may help people achieve informed choice, but primary care providers were found to be reserved in effectuating a carrier test if ethnicity was the only risk-factor presented by the patients. If the primary care providers felt that their colleagues were in favour of carrier testing solely on the basis of ethnicity, they might be prepared to act accordingly themselves. In order to prevent informed patients from being turned down for testing by their primary care providers, it is of great importance to motivate the primary care providers. If policy is to be developed towards facilitating testing, this study suggests that primary care providers, particularly GPs, should be targeted through opinion leaders and/or their peer organisations. At the same time, whole-system, participatory projects as described by Thomas et al. should also be tried [28].

## Competing interests

The authors declare that they have no competing interests.

## Authors' contributions

SSW performed the statistical analysis and drafted the manuscript. ESLdeLK and FR advised on statistical methodology. MCC reviewed versions of the article and suggested revisions. MdK participated in the conception of the project and coordinated it. AMCP conceived of the questionnaire study and helped draft the manuscript. All authors read and approved the final manuscript.

## Pre-publication history

The pre-publication history for this paper can be accessed here:



## Supplementary Material

Additional file 1 **Questionnaire for high-risk group**. The file contains a detailed description of the questionnaire used for the high-risk group. Click here for file

Additional file 2 **Questionnaire for health care providers**. The file contains a detailed description of the questionnaire used for health care providers. Click here for file
