# Supplementary material for: Raising awareness of carrier testing for hereditary haemoglobinopathies in high-risk ethnic groups in the Netherlands: a pilot study among the general public and primary care providers
Source: BMC Public Health. 2009 Sep 15;9:338. doi: 10.1186/1471-2458-9-338 (PMC2754459; doi:10.1186/1471-2458-9-338)
Supplement: Additional file 1 — Questionnaire for high-risk group. The file contains a detailed description of the questionnaire used for the high-risk group. [file 1471-2458-9-338-S1.doc]

**Additional file 1. Questionnaire for high-risk group**

Unless otherwise stated, all items were measured on a 5-point scale.

**Measures**

*awareness and understanding*

Awareness was measured with three questions, each introduced with ‘Before you attended this session had you ever heard of...’ followed by ‘sickle cell disease or thalassaemia’,... ‘carriership for these diseases’ or ‘carriership testing for these diseases’. A response had to be chosen among among No, I had never heard of these diseases, Yes, I had heard of sickle cell disease, or Yes, I had heard of thalassaemia. The responses for the other two awareness questions substituted the following for the underlined words: carriership for these diseases or carriership testing for these diseases.

Understanding inheritance of haemoglobinopathies was tested with three statements: ‘A carrier of sickle cell disease or thalassaemia can also get sickle cell disease or thalassaemia’ and ‘You can be a carrier of sickle cell disease and/or thalassaemia if the disease doesn’t occur in your family’, with responses of Yes, I agree, No, I disagree, or I don’t know. The third statement was ‘Parents have a highly increased chance of having a child with sickle cell disease and/or thalassaemia if...’, to be completed with one of the following statements: both parents are carriers for the same disease, if only one of the parents is a carrier, or I don’t know. Responses were scored as correct (1) or unknown/incorrect (0).

*attitude towards information and participation*

Attitude towards information about carriership testing was measured by responses to the stem ‘I think information about carriership and carrier testing for sickle cell disease and/or thalassaemia is...’ through eight word pairs (good-bad, important-unimportant, reassuring-alarming, sensible-unwise, desirable-undesirable, pleasant-unpleasant, a privilege-discriminatory, beneficial-harmful).

Attitude towards participation in carrier testing was measured and scored in the same way, in response to the stem ‘I think participating in a carriership test for sickle cell disease and/or thalassaemia is...’ The items were recoded to a numerical value in a range of 1-5. A score of 5 indicated a positive attitude and a score of 1 indicated a negative attitude.

*social norm*

The social norm regarding participation in carrier testing was measured using two items: ‘I think my family thinks that I should have a carrier test’ (disagree-agree), and ‘I think my friends/acquaintances think that I should have a carrier test (disagree-agree)’ A score of 5 indicated social influence being of high importance positive attitude and a score of 1 indicated social influence being of practically no importance.

*perceived behavioural control*

Perceived behavioural control with regard to participation in carrier testing was measured using 4 items: ‘I am unable to get tested if blood will have to be drawn (disagree-agree)’; ‘I don’t think my general practitioner will effectuate carrier testing if I request it (disagree-agree)’;’Getting a carrier test is too time consuming (disagree-agree)’;’I would dread getting a carriership test (disagree-agree)’. The items in this scale were recoded. Within the range of 1-5, a score of 5 indicated the highest feeling of control and a score of 1 indicated the lowest feeling of control.

*intention*

Intention was measured in two ways: directly and indirectly, both through a single item; respectively ‘I want to find out for sure whether I am a carrier for sickle cell disease and/or thalassaemia (disagree-agree)’, and ‘I want to prevent the birth of a child with sickle cell disease and/or thalassaemia (disagree-agree)’.

*additional questions*

Further questions were posed about equal access to care, burden of carriership (stigmatization), risk perception, and possible future consequences of carriership.

*demographic variables*

Demographic variables assessed were sex, birth year, marital status, highest completed level of education, number of children, current pregnancy (of partner), country of birth, and country of birth of parents. Education was categorized as low (none, primary or pre-vocational secondary), intermediate (other secondary including senior vocational secondary) or high (vocational college or university). Ethnicity was coded by the following algorithm: by country of birth if not the Netherlands, if the Netherlands then by country of birth of mother, if the subject and mother were both born in the Netherlands then by country of father. The municipality of Amsterdam also uses this algorithm for demographic research, although the term ethnicity has recently been abandoned in favor of the term background (Nieuwe definitie allochtonen in Amsterdam [New Definition of Non-Native People in Amsterdam], Municipality of Amsterdam, Department of Research and Statistics, 2006).
