# Supplementary material for: Raising awareness of carrier testing for hereditary haemoglobinopathies in high-risk ethnic groups in the Netherlands: a pilot study among the general public and primary care providers
Source: BMC Public Health. 2009 Sep 15;9:338. doi: 10.1186/1471-2458-9-338 (PMC2754459; doi:10.1186/1471-2458-9-338)
Supplement: Additional file 2 — Questionnaire for health care providers. The file contains a detailed description of the questionnaire used for health care providers. [file 1471-2458-9-338-S2.doc]

**Additional file 2. Questionnaire for health care providers**

Unless otherwise stated, all items were measured on a 7-point scale.

**Measures**

*attitude*

Attitude was measured with responses to ‘What do you think of the current educational initiatives towards recommending a haemoglobinopathy carrier test solely on the basis of ethnicity?’ through six word pairs (*good-bad, important-unimportant, pleasant-unpleasant, desirable-undesirable, beneficial-harmful, a privilege-discriminatory*). Items were recoded to a numerical value in the range of 1-7, so that a score of 7 indicated a positive attitude towards educating patients about the subject, and a score of 1 indicated a negative attitude.

*social norm*

Social norm was measured by the question ‘Do you think your colleagues feel that patients should be referred for haemoglobinopathy carrier testing solely on the basis of ethnicity? *(certainly not-certainly)’*. All other determinants were measured through one single item.

*perceived behavioural control*

Perceived behavioural control was assessed through ‘Do you feel that you are actually able, at present, to refer every patient who wants to be tested for haemoglobinopathy carriership solely because of his or her ethnicity? *(certainly not-certainly)*’.

*current behaviour*

Current behaviour for effectuating carrier testing was assessed by the questions ‘Do you refer patients who request haemoglobinopathy carrier testing solely on the basis of ethnicity, without a family history of haemoglobinopathy? (never-always)’ and ‘Do you refer anaemic patients for haemoglobinopathy carrier testing solely on the basis of ethnicity (without a family history)? (never-always)’.

*intention*

Intention was measured by ‘Do you plan to refer patients for haemoglobinopathy carrier testing solely on the basis of ethnicity in the future? *(certainly not-certainly)*’.

*Opinion about policy*

Opinion about policy was assessed with a yes/no question posed as ‘Do you think standard referral of patients for haemoglobinopathy carrier testing, solely on the basis of ethnicity, should become policy?’
